# Papillomaviruses: a systematic review

**DOI:** 10.1590/1678-4685-GMB-2016-0128

**Published:** 2017-02-16

**Authors:** Rodrigo Pinheiro Araldi, Suely Muro Reis Assaf, Rodrigo Franco de Carvalho, Márcio Augusto Caldas Rocha de Carvalho, Jacqueline Mazzuchelli de Souza, Roberta Fiusa Magnelli, Diego Grando Módolo, Franco Peppino Roperto, Rita de Cassia Stocco, Willy Beçak

**Affiliations:** 1 Laboratório de Genética, Instituto Butantan, São Paulo, SP, Brazil; 2 Programa de Pós-graduação Interunidades em Biotecnologia, Instituto de Ciências Biomédicas (ICB), Universidade de São Paulo (USP), São Paulo, SP, Brazil; 3 Programa de Aprimoramento Profissional (PAP), Instituto Butantan, São Paulo, SP, Brazil; 4 Dipartimento di Medicina Veterinaria e Produzioni Animali, Università degli Studi di Napoli Federico II, Napoli, Campania, Italy

**Keywords:** bovine papillomavirus (BPV), bovine papillomatosis, carcinogenesis, natural history

## Abstract

In the last decades, a group of viruses has received great attention due to its relationship with cancer development and its wide distribution throughout the vertebrates: the papillomaviruses. In this article, we aim to review some of the most relevant reports concerning the use of bovines as an experimental model for studies related to papillomaviruses. Moreover, the obtained data contributes to the development of strategies against the clinical consequences of bovine papillomaviruses (BPV) that have led to drastic hazards to the herds. To overcome the problem, the vaccines that we have been developing involve recombinant DNA technology, aiming at prophylactic and therapeutic procedures. It is important to point out that these strategies can be used as models for innovative procedures against HPV, as this virus is the main causal agent of cervical cancer, the second most fatal cancer in women.

## A brief history of the papillomavirus (PVs) on carcinogenesis

In the last decades, novel diagnostic methods and therapies have been implemented in an attempt to combat cancer. However, the number of patients that succumb to the disease has increased globally (Varga *et al.*, 2014). This negative result emphasizes the complexity of the oncogenic process, which has a multifactorial cause. Among the etiological factors associated to cancer are the infectious agents, such as bacteria and viruses.

The association between cancer and infectious agents has been discussed for centuries (Graner, 2000). In 1858, George B. Wood stated in his book *Practice of Medicine* that cancer could be disseminated as an infectious disease (Graner, 2000). However, the association between cancer and infectious agents was only implied in the second half of 19^th^ century by Rudolf Maier (Graner, 2000; zur Hausen, 2009). The major difficulty in demonstrating this association can be attributed to the time-lapse of 15-40 years between the infection and the development of the first clinical signs that would allow cancer diagnosis (zur Hausen, 2009). Yet, in the last decades, the involvement of infectious agents with cancer has aroused great attention, as one in ten human malignancies is caused by these pathogens (Ribeiro-Müller and Müller, 2014).

Current studies estimate that 23% of all human malignancies are associated with infectious agents (zur Hausen, 2009; WHO 2013; Brücher and Jamall, 2014; Bravo and Felez-Sanchez, 2015). Among them, human papillomavirus (HPV) is responsible for 27.9% (zur Hausen, 2009) to 30.0% (Bravo *et al.*, 2010) of all incident cancer cases in the world. This data is very concerning, once 75% of the global population lives in developing countries (Marrazzo and Holmes, 2013), where the lack of information about the HPV and others sexually transmitted diseases (STD) contributes to the increase of HPV-associated cancers. According to the World Health Organization (WHO) (http://www.who.int/mediacentre/factsheets/fs380/en/), 85% of cervical cancer cases occurs in less developed countries.

Papillomaviruses (PVs) are not only associated to human cancers, but also with animal malignancies. Although there is no epidemiological data about the number of PV-associated incident animal cancers, this association is recognized since 1932 (Shope and Hurst, 1933; Graner, 2000). Moreover, veterinary research demonstrates an increase in both benign and malignant tumors (Misdorp, 1996), particularly in domestic animals (cats and dogs). Furthermore, animal neoplasms are important models for the study of human oncogenic process (Misdorp, 1996), by allowing the identification of molecular mechanisms associated to carcinogenesis (Cotchin, 1962, 1976) and novel therapeutics (Misdorp, 1996), and emphasizing the importance of comparative oncology.

In this review, we summarize relevant data and advances in papillomaviruses biology, including viral evolution, pathogenic mechanism of viral proteins and oncoproteins, ways of transmission, pathogenesis and oncogenesis. We also discuss the importance of BPV as a study model for HPV-associated oncogenic process.

## Genome organization of PVs

**Figure 1:**
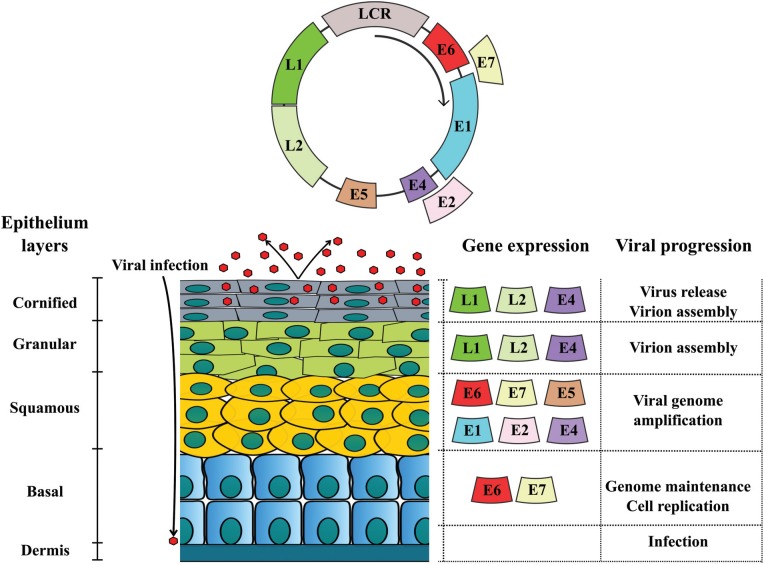
BPV genome organization and differential protein expression: double strand DNA circular genome divided in early (E), late (L) and long codon region (LCR). Early region codifies replication proteins (E1, E2, E4, E5, E6 and E7). These proteins are expressed from basal to cornified layer, being involved with virus replication and virion release (E4). Late region codifies L1 and L2 capsid proteins. Once these structural proteins are involved with virus assembly, they are expressed in the most differenciated epithelium layers (granular and cornified).

PVs are small, circular, double-stranded DNA viruses, able to infect all vertebrates (zur Hausen, 2009), as shown in Table 1. PVs belong to *Papillomaviridae* family (Bravo and Felez-Sanchez, 2015), which presents tropism for epithelial and mucous tissue (Cotchin, 1962). These viruses have genomes between 6,953 bp (CmPV1 - *Chelonia mydas* papillomavirus type 1) to 8,607 bp (CRPV1 - *Cotton rabbit* papillomavirus type 1), divided in three regions: early (E), late (L) and long codon region (LCR), as showed in Figure 1 (van Doorslaer, 2013). The E region codifies replication proteins (E1, E2, E4), and the oncoproteins E5, E6 and E7 (Bocaneti *et al.*, 2014). The L region codifies capsid proteins (L1 and L2) (van Doorslaer, 2013). The LCR does not codify any protein, but has the origin of replication (*ori*) (van Doorslaer, 2013). Currently, more than 280 different types of PVs are described. More than 200 types infect humans (Munday, 2014), which are classified in 49 genera according to the International Committee on Taxonomy of Viruses (ICTV) (Araldi *et al.*, 2015b). Phylogenetic classification of PVs are based on the L1 open reading frame (ORF) sequence homology, since this is the most conserved ORF among the different PV types (de Villiers *et al.*, 2004, de Villiers, 2013; Melo *et al.*, 2014; Munday *et al.*, 2015). According to this system, differences over 10% on L1 ORF sequence determine a novel virus type, while differences between 2-10%, a novel virus subtype (de Villiers, 2013).

**Table 1:** Papillomaviruses identified in different vertebrates

Species	Virus	References
Bovines	*Bos taurus* papillomavirus or Bovine papillomavirus (BPV)	Araldi *et al.* (2015b)
		Araldi *et al.* (2014a)
		Carvalho *et al.* (2013)
		Stocco dos Santos *et al.* (1998)
Canines	Canine oral papillomavirus (COPV) or *Cannis familiaris* papillomavirus (CfPV)	Nicholls *et al.* (2001)
		Zaugg *et al.* (2005)
Deer	*Capreolus capreolus* papillomavirus (CcPV) or *Cervus elaphus* papillomavirus (CePV)	Kràl *et al.* (2015)
Felines	*Felis catus* papillomavirus (FcaPV)	Munday (2014)
Rabbit	Cotton rabbit papillomavirus (CRPV)	Shope and Hurst (1933)
		Danos *et al.* (1985)
		Rashad and Evans (1967)
Raccoon	*Procyon loto* papillomavirus (PlPV)	Ng *et al.* (2015)
Seaotter	*Enhydralutris* papillomavirus (ElPV)	Ng *et al.* (2015)
Sheep	*Ovis aries* papillomavirus (OaPV)	Alberti *et al.* (2010)
Mice	*Mus musculus* papillomavirus (MmPV)	Handisurya *et al.* (2013)
Birds	*Fulmarus glaciallis* papillomavirus (FgPV)	Gaynor *et al.* (2015a)
	*Francolinusleucoscepus*papillomavirus (FlPV)	Moreno-Lopez *et al.* (1984)
	*Fringillacoelebs*papillomavirus (FcPV)	Van Bressem *et al.* (2007)
	*Psittacuserithacus*papillomavirus (PePV)	
Rat	*Rattus norvegicus* papillomavirus (RnPV)	Hansen *et al.* (2015)
Monkey	R*hesus macaca* papillomavirus(RmPV)	Roberts (2015)
Human	Human papillomavirus (HPV)	zur Hausen (2009)
		Herbster *et al.* (2012)

## Evolutionary history of papillomaviruses

Although the virus origin is still uncertain (Bernard, 1994; Holland and Domingo, 1998), studies about PVs suggest that they arose concomitantly with tetrapods in the Carboniferous period of the Paleozoic era (330 million years ago) (Rector and Van Ranst, 2013). This makes the PVs one of the oldest and largest known virus family (Cubie, 2013; Rector and Van Ranst, 2013).

Studies based on molecular phylogeny suggest that these viruses originated in Africa, from where they disseminated to all continents (Bernard, 1994). It was not a pandemic dissemination, occurring over one million years (Bernard, 1994).

PVs genomic diversification occurred together with mammals diversification, being influenced by multiple evolutionary forces (Bravo *et al.*, 2010), such as the addition of sequence boxes, previously present in their host (García-Vallvé *et al.*, 2005). Thus, PVs co-evolved with their respective host (Gottschling *et al.*, 2007). An evidence of this co-evolution is the similarity in guanine and cytosine (G + C) content; HPVs have 41-49% of G + C, and humans 40-42% (Black, 1968). Another evidence of this co-evolution is seen with the use of *in situ* hybridization probe for Shope papilloma virus (CRPV), which presents homology with the rabbit genome sequences (Black, 1968). Moreover, replicative mechanisms of PVs and their host are similar, reinforcing co-evolution (Leatherwood, 1998). These data suggest that PVs could have originated from genomic fragments of amniotes' common ancestor (van Doorlaer and Burk, 2010; Rector and Van Ranst, 2013; van Doorslaer, 2013).

Animal domestication favored the enzootic transmission of PVs (Gottschling *et al.*, 2007). In this process, novel strategies of adaptation were required to guarantee the infection of novel hosts (Gottschling *et al.*, 2011). These adaptations favored PVs evolution, conferring specificity in terms of host to all members of *Papillomaviridae* family, except for BPV, which is able to promote cross-infection (Bernard, 1994).

Based on this, García-Vallvé *et al.* (2005) described a hypothetical model to recreate PVs evolution. This model supports the existence of a proto-papillomavirus, comprised by URR-E1-E2-L2-L1 genomic regions, able to infect primitive amniotes. Along mammals divergence (150 million years ago), this proto-papillomavirus was added with E6 and E7 ORFs. From this moment, PVs' interaction with their host became more specific, resulting in a co-evolution. This process also resulted in the addition of E5 ORF in the hot spot between E2 and L2 ORFs (Bravo and Felez-Sanchez, 2015). Phylogenetic analyses point out that PVs only acquired oncogenic potential after they infected humans (García-Vallvé *et al.*, 2005). This suggests that BPV could have originated from HPV transmission to bovines, as a consequence of animal domestication (García-Vallvé *et al.*, 2005)., which would justify the similarities between these viruses, making the BPV a useful model to HPV studies (Campo, 2006; Borzacchiello and Roperto, 2008; Munday, 2014).

## Non-oncogenic early (E) proteins expressed by PVs: E1, E2 and E4

E1 and E2 ORFs are expressed after PV infection, once their genetic products are essential to virus replication (Ferraro *et al.*, 2011). The E1 ORF is the second most conserved sequence among the PVs (Forslund *et al.*, 1999; Enemark *et al.*, 2000). and codifies the E1 protein, which has three functional domains: (1) N-terminal, that induces Cdk2 phosphorylation, (2) C-terminal, that acts as ATP-dependent helicase and (3) central, that binds to E2 protein, resulting in E1-E2 complex (García-Vallvé *et al.*, 2005; Wallace and Galloway, 2014). The E1-E2 complex binds to viral replication origin (*ori*) present in LCR (Enemark *et al.*, 2000; Schuck and Stenlund, 2015). Next, the E1 protein forms the di-hexameric complex in *ori* (Gauson *et al.*, 2016), and recruits replication proteins such as topoisomerase I, DNA polymerase α and replication protein A (RPA), that need to replicate (Schuck and Stenlund, 2015). Due to its helicase activity, the E1 protein can induce simple strand breaks (SSBs) and double strand breaks (DSBs) in host DNA (Schuck and Stenlund, 2015).

E2 is a modular protein, composed by two domains: (1) C-terminal and (2) transactivation N-terminal (Wallace and Galloway, 2014). The E2 C-terminal binds to Brd4 protein (Gauson *et al.*, 2016). The E2-Brd4 complex interacts with lysine residues in acetylated histones (Schweiger *et al.*, 2006, 2007), resulting in an equitable distribution of virus copies after cytokinesis (Campo, 2006; Wallace and Galloway, 2014).

The E2 protein also acts as an E6 and E7 transcriptional regulator (García-Vallvé *et al.*, 2005; Bogaert *et al.*, 2008; Cai *et al.*, 2013). In high levels, E2 binds to 5′-ACCG(N)_4_CGGT-3′ palindromic sequence present in four E2 binding sites (E2BSs) in E6 and E7 promoter (P97) (Cai *et al.*, 2013). This inhibits RNA polymerase II binding, repressing E6 and E7 transcription (García-Vallvé *et al.*, 2005). However, under low expression levels, E2 recruits transcription factors necessary to form the open transcription complex (Helfer *et al.*, 2014; Jang *et al.*, 2014). In addition, E2 is an important epigenetic regulator, since the protein interacts with the p300/CBP-p/CAF global transcription activator (Wallace and Galloway, 2014). This interaction leads to *TP53* hypo-acetylation, reducing p53 expression levels (Wallace and Galloway, 2014).

The E4 ORF codifies a family of proteins produced by splicing followed by post-translational modifications (Campo, 1997a). The E4 protein is the most expressed protein of PVs (Doorbar, 2013). For this reason, E4 is easily detected in suprabasal and granulosum layers of epidermis (Rampias *et al.*, 2013), being recognized as important hallmark of PVs pathogenic activity (Doorbar, 2013). The E4 protein interacts with cytokeratin filamentous, contributing to viral replication (Campo, 1997a). Moreover, E4 is associated to virus maturation and extracellular matrix (MEC) remodeling (Ferraro *et al.*, 2011).

## Late proteins: L1 and L2

The L1 ORF is the most conserved among PVs (Bernard *et al.*, 2006; Haga *et al.*, 2013), and for this reason it is employed in virus classification (Haga *et al.*, 2013; de Villiers, 2013). The L1 protein has 55 kDa (Buck *et al.*, 2,013). and is able to self-organize in pentameric structures that compose the viral capsid (Ribeiro-Müller and Müller, 2014). It has a central role in viral infection mechanism, allowing the capsid anchorage to heparin sulfate receptors present in cell membrane (Florin *et al.*, 2012). Considering that L1 is a late protein, it is expressed in the most differentiated epithelium layers (Buck *et al.*, 2004). Therefore, L1 immunodetection has been considered the main evidence of productive infection (Nasir and Reid, 1999; Costa and Medeiros, 2014; Melo *et al.*, 2015; Araldi *et al.*, 2015b), which is characterized by the virus assembly (Green, 1972).

The L2 protein has 64-78 kDa (Wang and Roden, 2013). The molecular weight variation is a consequence of post-translational modifications (Wang and Roden, 2013). During the assembly of PV particles, 2 binds to viral DNA, contributing to encapsidation and then to viral release (García-Vallvé *et al.*, 2005; Campo, 2006).

A third structural protein (L3) has been described as present exclusively in BPV-4 (Catroxo *et al.*, 2013). However, its function remains unclear.

## Oncogenic proteins expressed by PVs: E5, E6 and E7

### E5 oncoprotein

The E5 is the most studied BPV oncoprotein and its transforming potential is known since 1960 (Roberts, 2015). The E5 oncoprotein can induce both *in vivo* and *in vitro* transformation (Campo, 2006; Silvestre *et al.*, 2009; Rampias *et al.*, 2013; DiMaio, 2014). Furthermore, it is responsible for the fibrotropism verified in fibropapillomas and equine sarcoids (Chambers *et al.*, 2003a; DiMaio, 2014).

PV-1 E5 is a transmembrane protein (Costa and Medeiros, 2014), with 43-44 amino acids, characterized by the presence of a central hydrophobic region, which acts as a transmembrane domain (Burkhardt *et al.*, 1989; Tomita *et al.*, 2007; DiMaio, 2014). E5 also presents two cysteine residues in the C-terminal region that confers stability for the homodimer composed by two E5 monomers (DiMaio, 2014).

The E5 oncoprotein induces cell membrane composition and dynamic changes, affecting the Golgi complex (Burkhardt *et al.*, 1989; García-Vallvé *et al.*, 2005; Balcos *et al.*, 2008). This occurs because E5 is able to bind to the H^+^-ATPase vacuolar subunit (Burnett *et al.*, 1992), promoting Golgi inner membrane alkalinization (Krawczyk *et al.*, 2010). This leads to major histocompatibility complex II (MHC-II) heavy chain sequestration, conferring an evolutionary mechanism of immune evasion (Venuti *et al.*, 2011). In addition, the E5 oncoprotein inhibits the expression of MHC-I and cyclooxygenase (COX) (Borzacchiello, 2007; Tomita *et al.*, 2007; Venuti *et al.*, 2011). These mechanisms contribute to viral infection persistence.

The E5 oncoprotein can bind to ductin, a conexon component, inhibiting the gap junction formation and therefore cell communication (Campo, 1997a; Borzacchiello and Roperto, 2008). E5 also contributes to focal adhesion loss, affecting cell differentiation (Rampias *et al.*, 2013).

The E5 oncoprotein is able to bind to platelet-derived growth factor receptor beta (PDGFβR) (Chambers *et al.*, 2003a; DiMaio, 2014; Costa and Medeiros, 2014). PDGFβR is a tyrosine-kinase receptor present in the cell surface (Nicolas *et al.*, 2013). Under normal conditions, PDGFβR is activated by PDGF, which promotes receptor dimerization (DiMaio, 2014) and activation of different kinases, such as: A-cdk2, MAPK, JNK, PI3K and C-Src (Chambers *et al.*, 2003a; Borzacchiello, 2007). However, E5 binding to PDGFβR can lead to phosphatidylinositol-3-kinase (PI3K)-mediated Akt pathway activation (Costa and Medeiros, 2014). This activates the D3 cyclin, promoting cell cycle deregulation (Venuti *et al.*, 2011; Costa and Medeiros, 2014). The PI3K/Akt pathway activation also induces metabolic deregulation in the host cell (Vivanco and Sawyers, 2002; Hennessy *et al.*, 2005). PDGFβR activation also recruits pericytes, stimulating angiogenesis (Pietras and Ostman, 2010).

### E6 oncoprotein

E6 is a small oncoprotein with 151-158 (HPV) (Ristriani *et al.*, 2000; Boulet *et al.*, 2007) or 137 amino acids (BPV-1) (Tong *et al.*, 1998; Mazzuchelli-de-Souza *et al.*, 2013), without enzymatic activity (Tan *et al.*, 2012a). The E6 oncoprotein is characterized by the presence of a class I PDZ domain (PSD-95/Dlg/ZO-1), which is located in the C-terminal, and four PVs conserved motifs (Cys-X-X-Cys) (Chen *et al.*, 1997; Tong *et al.*, 1998; Nominé *et al.*, 2006; Boon *et al.*, 2015). The PDZ domain is found in BPV (Mischo *et al.*, 2013) and high-risk HPVs (Boon *et al.*, 2015) and can interact with different proteins, promoting loss of intercellular contact and cell polarity (Boon *et al.*, 2015).

The HPV E6 oncoprotein can form a trimeric complex with E6AP ubiquitin ligase and p53 (Zanier *et al.*, 2005, 2007; Tan *et al.*, 2012a). This complex is responsible for the proteolytic degradation of p53 (Zanier *et al.*, 2005; Liu and Baleja, 2008), which directs the p53 to 26S proteasome (Cai *et al.*, 2013). The HPV-5 and 8 as well as BPV E6 oncoprotein interact with the CBP/p300 complex (Werness *et al.*, 1990; Zimmermann *et al.*, 2000; Liu *et al.*, 2005), promoting p53 downregulation (Zimmermann *et al.*, 1999). The loss of p53 contributes to genomic instability verified in cells infected by PVs (Araldi *et al.*, 2013, 2015b), as well as in cells treated with BPV-1 E6 recombinant oncoprotein (Araldi *et al.*, 2015a).

The overexpression of p53 represents a threat to viral replication (Shamanna *et al.*, 2013). For this reason, different oncogenic viruses express proteins able to promote p53 deregulation. Among these proteins are antigen T of SV40, adenovirus E1B, hepatitis B virus (HBV) HBx and PVs E6 (Shamanna *et al.*, 2013; Cuninghame *et al.*, 2014). Thus, p53 deregulation is a characteristic shared by oncogenic viruses.

The E6 protein can interact with paxillin (Turner, 2000; Zanier *et al.*, 2005) and AP-1 gamma subunit clathrin adapter (Tong *et al.*, 1998). These interactions can lead to cytoskeleton alterations, affecting the vesicular traffic. E6 can also interact with fibulin 1, contributing to invasiveness phenotype (Moody and Laimins, 2010).

Studies have also demonstrated that E6 oncoprotein can induce cell transformation (Liu *et al.*, 2002) and immortalization (Boon *et al.*, 2015) due to the up-regulation of telomerases (Cuninghame *et al.*, 2014). This occurs because E6 promotes *FOXM1* overexpression, resulting in cyclin B1, D1 and cdc25 expression and, therefore, cell proliferation (Chen and Lee, 2015). In addition, E6 is able to bind to LXXL motif of *MAML1* transcriptional regulator, inhibiting the *Notch* signaling (Tan *et al.*, 2012a; White and Howley, 2013). The E6 oncoprotein of both HPV and BPV also promotes energy metabolic deregulation, contributing to cell transformation (Araldi *et al.*, 2016).

E6 inhibits the SSB repair system, leading to genomic instability. This occurs because the oncoprotein interacts with XRCC1 and O^6^-methylguanosine-DNA-methyltransferase protein, which is recruited during the SSB repair (Wallace and Galloway, 2014). E6 also leads to cytogenetic damages (Wallace and Galloway, 2014) and stimulates neosis (Araldi *et al.*, 2015a).

### E7 oncoprotein

The E7 oncoprotein has 127 amino acids (Borzacchiello, 2007), and is able to bind to LXCXE conserved motif of pRb tumor suppressor protein (Moody and Laimins, 2010; White and Howley, 2013). This binding results in pRb phosphorylation, leading to E2F factor translocation to the nucleus (Longo, 2013). The E2F factor recruits different chromatin modifiers, including histone deacetylases (HDAC) (Moody and Laimins, 2010). Thus, the E7 oncoprotein induces the constitutive expression of E2F-responsive genes, such as cyclin A and E (Moody and Laimins, 2010), leading to S and G2/M cell cycle phase increase (Sacco *et al.*, 2003; Ferraro *et al.*, 2011). The interaction between viral oncoproteins and pRb is not exclusive of PVs, being also observed with adenovirus E1A and antigen T of SV40 (White *et al.*, 2015).

Studies have demonstrated that E7 induces DNA breaks, contributing to cell cycle deregulation (Park *et al.*, 2014; Araldi 2015; Araldi *et al.*, 2015b). In addition, the E7 oncoprotein increases the *Sirt1* deacetylase expression levels, modulating *γ*-H2AX activity (Park *et al.*, 2014), a protein that participates in DSB repair (Sedelnikova and Pilch, 2003; Chowdhury *et al.*, 2005). Thus, the E7 oncoprotein inhibits DSB repair, resulting in genomic instability.

## Bovine papillomavirus (BPV): first reports and questions

In 1986 in an intriguing paper by Campo and Jarret (1986) reported the description of six different BPV types (BPV-1 to BPV-6) classified into two subgroups: subgroup A, that promotes fibropapillomas, and subgroup B, which leads to true epithelial papillomas. The report also showed that the BPV-4, a member of subgroup B, was the etiological agent of papillomas of the upper digestive tract, which could become carcinomas in animals feeding on a specific bracken fern pasture (*Pteridium aquilinum*). Later, Walter-Moura *et al.* (1988) verified the increase of chromosome aberrations in cells obtained from short term peripheral lymphocytes collected from bovines afflicted with bovine enzootic hematuria (BHE) that were exposed to pastures with bracken fern. These studies were reinforced by following reports, demonstrating that carcinogenesis is associated to the interaction between BPV and carcinogens present in the fern, such as quercetin and ptaquilosides (Pennie and Campo, 1992; Shahin *et al.*, 1999; Potter and Baird, 2000; Beniston *et al.*, 2001; Leal *et al.*, 2003; Lioi *et al.*, 2004). These studies brought new questions to be answered: how can the co-factor act in synergism with the virus? Why can these effects be detected in peripheral blood, considering that the virus is epithelial? Or, could the effect on chromosomes be only related to bracken fern compounds?

## BPV and bovine papillomatosis (BP)

BPV is a cosmopolitan virus, worldwide distributed, independently of the level of expertise on livestock exploration (He *et al.*, 2014). It is estimated that 60% of Brazilian cattle herd is infected by BPV (Stocco dos Santos, *et al.*, 1998). However, that rate can be higher, once virus infection can be asymptomatic (Araldi *et al.*, 2013; Silva *et al.*, 2013a). Furthermore, the absence of epidemiological studies about BPV distribution could underestimate the real percentage of infected animals, representing a notorious difficulty in attempting to develop vaccines, once we do not know the prevalent virus types in each country.

**Figure 2:**
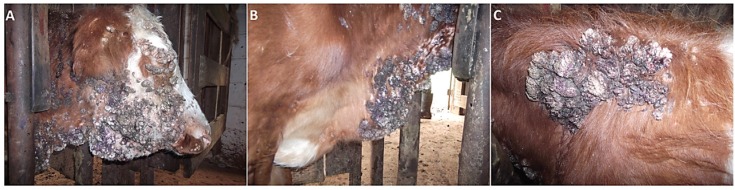
Adult bovine (Simmental breed) with bovine papillomatosis (BP): skin papillomas on face (A), dewlap (B) and back (C).

BPV infection is endemic in both dairy and beef cattle breeding (Claus *et al.*, 2009; Araldi, 2015,; Araldi *et al.*, 2015b). However, BPV presents a predilection for dairy cattle (Araldi, 2015). The virus is the causal agent of bovine papillomatosis (BP) (Muro *et al.*, 2008), an infectious, contagious and neoplastic disease, characterized by the presence of multiple benign tumors (papillomas) (Figure 2) that can regress spontaneously or progress to malignant neoplasms (Campo, 2002; Turk *et al.*, 2005; Yaguiu *et al.*, 2006; Borzacchiello and Roperto, 2008; Araldi *et al.*, 2014b; Bocaneti *et al.*, 2014). Although BP affects preferably young cattle, the disease can occur at all ages (Börkü *et al.*, 2007). The persistence of papillomas can lead to feeding and breathing difficulties, requiring the animal's euthanasia (Campo, 2002). Moreover, BP decreases growth rate and induces weight loss (Munday, 2014). The disease also predisposes bovines to opportunist bacterial infections (Munday, 2014), mainly in breast and mammary glands, which can result in mastitis, causing pain, reducing milk production (Campo, 2002; Muro *et al.*, 2008; Lindsey *et al.*, 2009; Santos *et al.*, 2014) and causing depreciation of leather value (Monteiro *et al.*, 2008).

Cutaneous papillomas are proliferative benign tumors, with complex pathogenesis (Börkü *et al.*, 2007). These neoplasms commonly arise in vascularized areas exposed to physical attrition (Özsoy *et al.*, 2011). Histologically, papillomas are characterized by epithelium hyperplasia, showing an enlargement of interpapillary ridge that extend above dermis (Monteiro *et al.*, 2008). Morphologically, they are classified in: (1) typical - exophytic masses, with “cauliflower” aspect, presenting a wide or narrow insertion base, (2) pedunculated - exophytic masses connected by a narrow base with a peduncle shape, (3) atypical or flat - dense and flat exophytic masses entirely connected with the tissue, (4) filamentous - exophytic masses with highly keratinized surface and a thin base, present in mammary glands and (5) rice-form - small papillomas with rice-like shape (Monteiro *et al.*, 2008).

Currently, 15 types of BPV are described (Munday *et al.*, 2015), and classified in four genera: *Deltapapillomavirus* (BPV-1, 2, 13 and 14), *Epsilonpapillomavirus* (BPV-5 and 8), *Xipapillomavirus* (BPV-3, 4, 6, 9, 10, 11, 12 and 15) and *Dyoxipapillomavirus* (BPV-7) (Melo *et al.*, 2014; Grindatto *et al.*, 2015; Munday *et al.*, 2015; Silva *et al.*, 2016). *Delta* and *Epsilonpapillomavirus* are associated with both papillomas and fibropapillomas, while *Xipapillomavirus*, only to squamous papillomas (Tomita *et al.*, 2007; Yuan *et al.*, 2007; Tan *et al.*, 2012b; Araldi, 2015; Araldi *et al.*, 2015b).

Although there is no epidemiological study that allows the definition of BPV distribution, BPV-1 and 2 seem to be the most frequently identified virus types (Pathania *et al.*, 2012; Melo *et al.*, 2014; Santos *et al.*, 2014; Alcântara *et al.*, 2015; Araldi, 2015; Cota *et al.*, 2015; Araldi *et al.*, 2015b). Moreover, these viruses are associated with both benign and malignant neoplasms (Gopalkrishna *et al.*, 1995; Nasir and Campo, 2008; Cota *et al.*, 2015). However, the global distribution of BPV is not homogenous (Santos *et al.*, 2014). BPV-1 and 2 have closely related serotypes (Shafti-Keramat *et al.*, 2009), associated with urinary bladder malignant neoplasms (Wosiacki *et al.*, 2005; Balcos *et al.*, 2008; Roperto *et al.*, 2008; Maiolino *et al.*, 2013; Cota *et al.*, 2015). BPV-4 infection is an important cause of upper digestive tract cancer development (Tsirimonaki *et al.*, 2003; Nasir and Campo, 2008; Lucena *et al.*, 2011). Studies also show that BPV-13 is associated to urothelial carcinomas (Roperto *et al.*, 2015).

## Diagnostic methods

Studies about BPV diversity and prevalence are mandatory to develop novel therapeutic methods (Silva *et al.*, 2013b), since immunity is species-specific (Claus *et al.*, 2007). Therefore, diagnosis is crucial.

Different methods have been implemented to identify PVs, such as: *Southern blot* (Leto *et al.*, 2011), immunohistochemistry (IHC) (Elzein *et al.*, 1991; Araldi *et al.*, 2015b), chromogenic *in situ* hybridization (CISH) (Melo *et al.* 2015), electron microscopy (Araldi *et al.*, 2014b) and PCR using specific and/or degenerate primers (Stocco dos Santos *et al.*, 1998; Araldi *et al.*, 2013, 2014a, 2015b; Melo *et al.*, 2014). Among these techniques, PCR is the most used due to its high sensitivity (Leto *et al.*, 2011). Moreover, using the restriction fragment length polymorphism of PCR products (PCR-RFLP) allows to identify BPV type (Carvalho *et al.*, 2013), since this method shows a correlation of 95% with the results obtained using DNA sequencing (Martens *et al.*, 2001a; Carvalho *et al.*, 2013; Kawauchi *et al.*, 2015).

Although real-time PCR (qRT-PCR) allows to determine the number of viral copies, this method has the lowest reproducibility (Guo *et al.*, 2012). For this reason, PCR followed by DNA sequencing represents the most common method to identify and typify BPVs (Melo *et al.*, 2014; Araldi *et al.*, 2014b). However, there is no “gold-standard” primer employed in PVs identification (Antonsson *et al.*, 2010).

On the one hand, although the specific primers have higher sensitivity than degenerate primers, they cannot identify the 14 BPV types simultaneously (Araldi *et al.*, 2014b). In addition, specific primers do not allow to identify novel virus types and subtypes (Araldi *et al.*, 2014b). Moreover, evidence indicates that a specific primer for BPV-1 can anneal to BPV-2 (Haga *et al.*, 2013), once these virus types are considered serotypes-like (Shafti-Keramat *et al.*, 2009). However, in a comparative study using complete genomes of BPV-1-6 in 2014 we demonstrated the BPV-1 primer specificity (Araldi *et al.*, 2014b).

Among the degenerate primers described in the literature, FAP59/64 (Forslund *et al.*, 1999) is the most employed in both BPV and HPV identification. This primer was designed based on the L1 ORF homology of HPV (Forslund *et al.*, 1999) and later optimized to identify BPV DNA sequences (Ogawa *et al.*, 2004). Furthermore, the use of FAP59/64 primer already allowed to identity novel BPV types, including BPV-9 and 10 (Hatama *et al.*, 2008). However, despite of these advantages of degenerate primers, there are several reports showing their low sensitivity. For instance, MY09/11, another degenerate primer frequently used in HPV diagnosis, was unable to identify BPV and HPV sequences in clinical samples (Martelli-Marzagão *et al.*, 2010; Zhu *et al.*, 2014) or copies of complete cloned genome (Araldi *et al.*, 2014b).

Although PCR is commonly used to identify PV DNA sequences, the method does not allow to identify their localization and physical state (episomal or integrated). Therefore, CISH represents and additional method used to demonstrate the physical state of these viruses (Black, 1968; Munday, 2014; Melo *et al.*, 2015). Another additional technique to identify PVs is L1 immunodetection (Nasir and Reid, 1999; Longworth and Laimins. 2004; Roperto *et al.*, 2011; Munday, 2014; Melo *et al.*, 2015; Araldi *et al.*, 2015b)., which not only allows identification of the viral presence, but also provides an important evidence of productive infection (Nasir and Reid, 1999; Roperto *et al.*, 2011; Melo *et al.*, 2015).

Histopathological analysis of BPV-infected lesions is a differential diagnosis method, which allows to identify intra-epithelial neoplasms with oncogenic potential (Monteiro *et al.*, 2008; Araldi *et al.*, 2015b).

## BPV infection pathway and histopathological alterations

BPV transmission can occur by direct (animal-animal) or indirect contact with contaminated surfaces (Muro *et al.*, 2008; Cubie, 2013). Studies also show that the virus can be transmitted by flies (Finlay *et al.*, 2009) and ticks (Muro *et al.*, 2008).

Studies of PV infection are based on the prototype BPV-1 (Florin *et al.*, 2012). According to the literature, the viral infection requires a tissue micro-injury, exposing heparin sulfate proteoglycan receptors (Ljubojevic and Skerlev, 2014; Greber, 2016), which are needed for BPV L1 anchorage (Hartl *et al.*, 2011; Buck *et al.*, 2013)., This is confirmed by treatment with heparinase preventing viral infection (Kines *et al.*, 2016). The micro-injury is necessary for the virus to access basal keratinocytes (Liu and Baleja, 2008), where the virus cycle begins (Campo, 1997a). Current studies also show that integrin α6 (CD49f) and integrin 332 (laminin 5) are targets for L1 binding (Sibbet *et al.*, 2000; Florin *et al.*, 2012).

The L1 binding to heparin sulfate leads to conformational changes in capsid icosaedric structure (Buck *et al.*, 2013). This exposes the L2 N-terminal to be cleaved by furin protein, present in the cell membrane (Buck *et al.*, 2013). This cleavage induces a second capsid conformational change, allowing L2 to bind to different receptors, such as integrin α2β4 (Buck *et al.*, 2013). Next, the virions are internalized by an clathrin-dependent endocytose mechanism, resulting in cytoplasmic vesicles that associate to lysosomes (Day *et al.*, 2003). The lysosomal acid content release promotes pH alterations in capsid proteins, resulting in viral DNA release (Day *et al.*, 2003). The BPV genome is found in epissomal form (Campo, 2002; Munday, 2014; Cota *et al.*, 2015), while HPV can integrate in fragile sites of the host genome (Monte and Peixoto, 2010; Moody and Laimins, 2010; Munday, 2014). A current study based on qRT-PCR, showed that cutaneous papillomas have about 2.210^4^ viral copies (Cota *et al.*, 2015).

PVs do not codify polymerase (Moody and Laimins, 2010). For this reason, these viruses induce the S-phase entry, which was verified in BPV-infected cells (Potocki *et al.*, 2014), in a process known as amplification (Moody and Laimins, 2010). Due to the stimulation of cell proliferation, BPV induces mitotic stress (Potocki *et al.*, 2014), resulting in cytogenetic aberrations (Stocco dos Santos *et al.*, 1998; Melo *et al.*, 2011; Araldi *et al.*, 2013; Campos *et al.*, 2013). Moreover, the viral hyperproliferative action leads to the exophytic mass development as a consequence of acanthosis (Cubie, 2013; Munday, 2014; Araldi *et al.*, 2015b). As occurs with epithelium differentiation and virus assembly, a keratinization process is verified (Ferraro *et al.*, 2011). This process is histologically characterized by the increase of keratin granules in the granular layer (Ferraro *et al.*, 2011; Araldi *et al.*, 2015b).

Virus assembly is observed in the most differentiated epithelium layers (Munday, 2014), where the virion release occurs by cell degeneration (Brobst and Hinsman, 1966; Buck *et al.*, 2013). This process results in koilocyte formation. The term koilocyte comes from the Greek word *koillos*, which means “cavity” (Ferraro *et al.*, 2011). Koilocyte formation results from the cytopathic effect of E5 and E6 oncoproteins, although the molecular mechanism that results in cell vacuolization remains unclear (Krawczyk *et al.*, 2008). However, the cytoplasmic vacuolization contributes to cell fragility and virion release (Krawczyk *et al.*, 2008; Wang and Kieff, 2013). In this sense, koilocytes are cells destined to apoptosis, which emerges as a consequence of DNA replication and macromolecule synthesis inhibition.

After viral assembly, virions are released in the corneum layer, allowing infiltration in the keratin matrix (Brobst and Hinsman, 1966; Buck *et al.*, 2013). This mechanism confers an immune evasion, since the icosaedric morphology of PVs is immunoreactive (Buck *et al.*, 2013). In addition, keratin confers physical protection for virions, as they are non-enveloped.

## BPV as a model for HPV

HPV is species-specific, infecting exclusively humans (Koller and Olson, 1972; Campo, 2002). This specificity was demonstrated in the 1970s, when calves, hamsters, ponies and Rhesus monkeys were inoculated with BPV and HPV virions isolated by ultracentrifugation (Koller and Olson, 1972). However, only BPV was able to infect the species (Koller and Olson, 1972). Due to its capability to infect different species and the pathogenic and morphological characteristics shared with HPV (Munday, 2014), BPV has been used as a prototype to study PV biology and oncology (Koller and Olson, 1972; Campo, 2002; Liu *et al.*, 2005; Campo, 2006; Costa and Medeiros, 2014). Therefore, research involving BPV has contributed with the understanding of viral oncogenesis (Costa and Medeiros, 2014; Munday, 2014). Based on these data, the next sections will focus on BPV and its host interaction.

## Equine sarcoid: an example of cross-infection

Although considered epithelial- and mucous-tropic, some BPV types, especially *Deltapapillomavirus*, can infect fibroblasts causing sarcoma-like lesions, known as equine sarcoid (Chambers *et al.*, 2003b; Geisshüsler *et al.*, 2016). Equine sarcoid was first described by Jackson (1936), being considered a biphasic neoplasia, since it affects both epidermis and dermis. Equine sarcoid is the most frequent benign skin neoplasia observed in horses that are 1-6 years old (Jackson, 1936; Otten *et al.*, 1993; Nasir and Reid, 1999; Martens *et al.*, 2000; Bergvall, 2013; Mosseri *et al.*, 2014), affecting 11.5% of all horses (Knottenbelt, 2005). Differently from papillomas, sarcoids rarely present a spontaneous regression (Angelos *et al.*, 1991).

Equine sarcoid is an invasive but not metastatic neoplasia (Nasir and Reid, 1999), causing substantial morbidity and economic loss due to aesthetic and functional impairment (Angelos *et al.*, 1991; Otten *et al.*, 1993). Six different sarcoid morphotypes are known: occult, verrucous, nodular, fibroblastic, mixed and malignant (Knottenbelt, 2005). However, 84% of affected equines have more than one sarcoid morphotype (Goodrich *et al.*, 1998).

Equine sarcoids are lesions with intense fibroblastic proliferation, in which fibroblasts are disposed in fusiform bundles or spirally organized, presenting a morphology of fibropapilloma-like (Martens *et al.*, 2000). Another characteristic of this neoplasia is the presence of anaplastic and pleomorphic fibroblasts, perpendiculary orientated in relation to basal membrane, and being observed in the dermo-epidermal junction (Bogaert *et al.*, 2010). The epidermal component is only present in verrucous and mixed sarcoids (Bogaert *et al.*, 2010). The sarcoid invasiveness capability can be attributed to the high levels of expression of metalloproteinase. MMP1 promotes the laminin and collagen IV degradation, resulting in extracellular matrix remodeling (Mosseri *et al.*, 2014)

Equine sarcoid has a multifactorial cause (Bergvall, 2013). However, the *Deltapapillomavirus* is recognized as an etiological factor (Nasir and Reid, 1999; Chambers *et al.*, 2003b; Bogaert *et al.*, 2008b; Bergvall, 2013). The association between BPV and equine sarcoid was first described by Olson and Cook in 1951 (Brandt *et al.*, 2008). BPV-1 and 2 DNA sequences are identified in 100% of equine sarcoids (Martens *et al.*, 2001a; Gaynor *et al.*, 2015b). Furthermore, DNA sequences of these virus types are identified in 2/3 of asymptomatic horses (Bravo *et al.*, 2010). These data demonstrate that the virus can be asymptomatic, remaining latent in epidermis and dermis (Bogaert *et al.*, 2008b; Brandt *et al.*, 2008; Bergvall, 2013).

Viral latency is a characteristic shared by BPV (Bogaert *et al.*, 2008b; Araldi *et al.*, 2013; Silva *et al.*, 2013a) and HPV (Maran *et al.*, 1995; Astori *et al.*, 1998; Forslund *et al.*, 2004). However, the virus infection induces DNA damages and genomic instability (Stocco dos Santos, *et al.*, 1998; Melo *et al.*, 2011; Araldi *et al.*, 2013; Campos *et al.*, 2013; Melo *et al.*, 2015). Moreover, studies show that 70% of BPV-infected asymptomatic horses live in contact with cattle (Bergvall, 2013). However, although the presence of BPV-1 L1 transcripts was already verified in equine sarcoids by RT-PCR (Nasir and Reid, 1999), these neoplasias are considered abortive infection sites, since virion presence was not described. (Bogaert *et al.*, 2008b; Brandt *et al.*, 2008). The absence of productive infection in equine sarcoids suggests that the BPV cross-infection is a consequence of an erratic virus cycle (Otten *et al.*, 1993).

As verified in PB, sarcoids are frequently seen in sites most susceptible to traumatism (Angelos *et al.*, 1991; Otten *et al.*, 1993; Martens *et al.*, 2000), confirming the need of tissue micro-injury for virus infection. In addition, as in bovines, it is believed that insects can contribute to BPV transmission, because BPV-1 DNA sequences were already identified in *Musca automnalis, Fannia carnicularis* and *Stomoxys calcinatrans* flies (Bergvall, 2013).

Studies also show that the Arabic breed is the most susceptible to sarcoid development (Knottenbelt, 2005; Bogaert *et al.*, 2008b). The reason for this is the presence of W3 and B1 MHC-II haplotypes that facilitate BPV infection persistence (Chambers *et al.*, 2003b; Bogaert *et al.*, 2008b).

Currently, treatments for sarcoid, as well as for PB, are almost inefficient (Bergvall, 2013). Treatment methods are: (1) surgical excision of the neoplasia, with recurrence in 50-64% of the cases within six months (Lancaster *et al.*, 1977; Martens *et al.*, 2001b; Bergvall, 2013; Mosseri *et al.*, 2014), (2) laser therapy, where recurrence is 38% (Martens *et al.*, 2001b); cryotherapy, which is inefficient for lesions larger than 2 cm^2^ (Carr, 2009), and chemotherapy using cisplatin or 5'-fluouracyl (5-FU), that can cause nephro and hepatotoxicity (Stewart *et al.*, 2006). The recurrence of disease is argued to be a consequence of BPV presence in the surgical margin (Martens *et al.*, 2001a). However, a current study showed a lack of correlation between BPV DNA in surgical margins and recurrence of equine sarcoids (Taylor *et al.*, 2014). Meanwhile, Brandt *et al.* (2008) identified BPV DNA sequences in peripheral blood of sarcoid-affected equines. These data suggest that, as verified in bovines (Stocco dos Santos *et al.*, 1998; Roperto *et al.*, 2011; Araldi *et al.*, 2013; Melo *et al.*, 2015), the peripheral blood can be argued as being a vehicle of viral dissemination.

## Breaking paradigms

After the first reports (Campo and Jarret, 1986; Walter-Moura *et al.*, 1988), our purpose of investigation was to examine the role of peripheral blood as a potential BPV-transmitting agent. The first data came from Stocco dos Santos *et al.* (1998), which showed high levels of chromosomal aberrations in lymphocytes infected by BPV-2. That study also described the presence of BPV-2 DNA sequences in peripheral blood of donor and recipient animals and in the progeny of recipient animals. These results were the first evidence of vertical transmission (Freitas *et al.*, 2003). In this study, we verified the presence of BPV DNA sequences and DNA damages in peripheral blood samples of animals from different areas of Brazil (Diniz *et al.*, 2009; Melo *et al.*, 2011; Araldi *et al.*, 2013). In addition, we also demonstrated the presence of different BPV types in peripheral blood and cutaneous papilloma (Araldi *et al.*, 2014a). All these data suggest a viral activity in blood cells (Stocco dos Santos *et al.*, 1998; Araldi *et al.*, 2013). The presence of BPV DNA sequences in peripheral blood mononuclear cells (PBMCs) was also described in the literature in both bovines (Roperto *et al.*, 2008; Silva *et al.*, 2013a) and equines (Brandt *et al.*, 2008, 2011), reinforcing our results. Following studies also showed the presence of BPV transcripts and the L1 capsid protein in PBMCs, demonstrating the productive infection in blood cells (Roperto *et al.*, 2011; Melo *et al.*, 2015). We also described the presence of BPV DNA sequences in different non-epithelial tissues such as spermatozoa, urine and milk (Lindsey *et al.*, 2009). These data demonstrate the need to review the natural history of papillomavirus, as currently proposed by Munday (2014).

**Figure 3:**
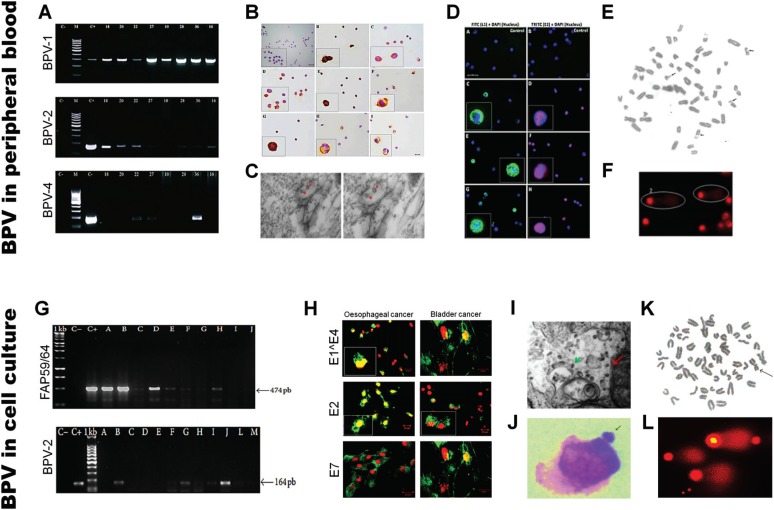
Evidences of BPV presence in peripheral blood and cell culture showing: A) amplicon of BPV identified using specific primers for BPV-1, 2 and 4 (Araldi *et al.*, 2013); B) BPV genome identification by chromogenic in situ hybridization (Melo *et al.*, 2015); C) BPV virions identified in cytoplasmic vesicles of PBMCs (Melo *et al.*, 2015); D) Immunodetection of BPV L1 and E2 in lymphocytes (Melo *et al.*, 2015); E) cytogenetic aberrations (breaks) observed in BPV-infected lymphocyte (Stocco dos Santos *et al.*, 1998), F) class 2 nucleoids observed in BPV-infected lymphocytes (Araldi *et al.*, 2013); G) BPV DNA sequences identified in cell culture, using FAP59/64 degenerate primers; H) Immunodetection of BPV early proteins (E1^E4, E2, E7) in both oesophageal and urinary bladder carcinoma cell line (Melo *et al*., data not published); I) BPV virion, with 55 nm, in cytoplasmic vesicle of cutaneous papilloma cell line, total magnification of 60000X (Melo *et al*., data not published); J) bovine epithelial cell line (CRIB) treated with E6 recombinant oncoprotein showing micronucleus presence (Araldi *et al.*, 2015a); K) cytogenetic aberration observed in BPV-infected cell line (Campos *et al.*, 2013); L) class 2 nucleoids observed in cutaneous papilloma cell line (Araldi et al., data not published).

We verified that primary culture cell lines from BPV-infected cutaneous and esophageal papilloma have chromosomal aberrations similar to those verified in peripheral blood (Campos *et al.*, 2013). In addition, using BPV-4 E7 oncoprotein transformed PALF cell lines, we demonstrated the mutagenic potential of quercetin, a flavonoid found in bracken fern *P. aquilinum,* which is recognized as a co-factor to BPV-associated upper gastric cancer (Leal *et al.*, 2003). In a current study, we also showed that the BPV induced metabolic alteration in host cells, increasing reactive oxygen species and DNA damages (Araldi, 2016; Araldi *et al.*, 2016). A summary of our main results is shown in Figure 3. These data suggest that BPV-infected cell lines are a useful model to study the pathogenic mechanisms that lead to cancer. Our contribution has allowed to know the BPV prevalence and distribution in Brazil (Diniz *et al.*, 2009; Carvalho *et al.*, 2013; Lunardi *et al.*, 2013; Melo *et al.*, 2014; Araldi *et al.*, 2014b; Cota *et al.*, 2015; Gaynor *et al.*, 2015a; Grindatto *et al.*, 2015; Dong *et al.*, 2016; Martano *et al.*, 2016). These studies showed that the co-infection of at least two BPV types is frequent (Araldi *et al.*, 2013, 2014a; Carvalho *et al.*, 2013), demonstrating the importance to develop multivalent vaccines.

## BPV-associated malignant neoplasms

### Urinary bladder carcinoma

Urinary bladder carcinoma represents 0.01% of all bovine cancers (Roperto *et al.*, 2015). It is estimated that urinary bladder cancer has caused economic losses of € 4 million between 2000-2006 in the Azores (Costa and Medeiros, 2014).

The etiopathogenic role of BPV in urinary bladder carcinoma was first described in 1955 in Brazil and South Africa (Plowrigh, 1955). Currently, the BPV promoter action in urothelial carcinoma is well established. This is because sequences of BPV-1, 2, 13 and 14, as well as the expression of E5 oncoprotein are detected in this neoplasms (Wosiacki *et al.*, 2006; Balcos *et al.*, 2008; Roperto *et al.*, 2016; Russo *et al.*, 2016).

Urinary bladder carcinoma is clinically characterized by bovine enzootic hematuria (BHE) (Wosiacki *et al.*, 2002), which is verified in more than 90% of cattle with urinary bladder cancer (Resendes *et al.*, 2011). BHE is characterized by intermittent hematuria, which can lead to anemia and weight loss (Wosiacki *et al.*, 2002). The disease affects preferably cattle between 3-5 years old, without breed preference (Wosiacki *et al.*, 2002). BHE is associated to dietary intake of carcinogenic compounds present in *P. aquilinum, P. esculetum, P. revolution, Chelanthes seiberi, Encephalartos hildebrandti, Ranunaelus montana, R. acris, Climatis vitalbi* (Wosiacki *et al.*, 2002). Among these species, *P. aquilinum* stands out for its wide biogeographical distribution (Oliveira, 2012). Bracken fern is commonly observed in the South of Brazil, where there is a high incidence of BEH (Dias *et al.*, 2012).

*P. aquilinum* presents high levels of immunosuppressor and carcinogenic compounds, including quercetin, ptaquilosides and shikimic acid (Shahin *et al.*, 1999; Beniston *et al.*, 2001; Bonadies *et al.*, 2004). The immunosuppressor activity of these metabolites contributes to BPV infection persistence and represents an additional source of DNA mutation (Leal *et al.*, 2003). According to Tokarnia *et al.* (2000), the diary intake of 10 kg of *Pteridum* ssp. in a year can lead to BEH. Moreover, the consumption of this bracken fern can result in urinary bladder and esophageal carcinoma (Masuda *et al.*, 2011).

Therefore, BPV-1 and 2 are considered important cofactors for BEH development (Campo, 1997a; Roperto *et al.*, 2005; Pavelski *et al.*, 2014), once BPV leads to epidermal and dermal hyperproliferation at the same time that it induces DNA damages, contributing to cancer initiation (Stocco dos Santos *et al.*, 1998; Araldi *et al.*, 2013; Campos *et al.*, 2013).

### Esophageal carcinoma (EC)

While the association between HPV and EC remains under discussion despite all evidences (Antonsson *et al.*, 2016), the etiological role of BPV in EC is well established (Borzacchiello *et al.*, 2003). EC in cattle is a self-limiting disease, being directly associated to BPV-4 infection (Borzacchiello *et al.*, 2003; Masuda *et al.*, 2011).

Although there is no report about BPV transmission in humans, a study performed in Germany showed a high incidence of warts in veterinarians that had contact with bovines (Bosse and Christophers, 1964). Another study in Central Asia discussed the association between milk consumption and EC (Nasrollahzadeh *et al.*, 2015). Considering that BPV DNA sequences were already detected in milk (Lindsey *et al.*, 2009) and the thermal resistance of viral capsid (Módolo *et al.*, data not published), it seems that the virus can survive to pasteurization process (zur Hausen, 2012). Therefore, more efforts are necessary to verify this possible cross-infection.

EC is the eighth most prevalent human malignancy, being considered the sixth *causa mortis* for cancer globally (Antonsson *et al.*, 2010; Herbster *et al.*, 2012). Considered the third most common gastrointestinal cancer (Felin *et al.*, 2008), EC has a high incidence in men (Bjørge *et al.*, 1997). In 2002, 462,000 novel cases of EC in the world were reported (Antonsson *et al.*, 2010). Brazil had 10,780 novel cases of EC in 2014 and 7,636 deaths due to the disease in 2011 (INCA, 2016).; its incidence has increased in the last years (INCA, 2016). This data leads to concern, since EC has a mortality rate that is 25% higher than cervical cancer (Han *et al.*, 1996; Kahrilas and Hirano, 2013).

Among the clinical signs of EC in humans are progressive dysphagia, weight loss, odynophagia, anorexia, fever and retrosternal pain (Felin *et al.*, 2008; Haster and Owyang, 2013), which are similar to those verified in bovines (Borzacchiello *et al.*, 2003). Scarce epidemiological data about EC in cattle are available. In humans, EC has a variable incidence according to geographic area (Bjørge *et al.*, 1997; Syrjänen, 2002; Guo *et al.*, 2012; Nasrollahzadeh *et al.*, 2015; Antonsson *et al.*, 2016). Among countries with high EC incidence are China, Singapore, Iran, South Africa and Brazil (Bey *et al.*, 1976; Han *et al.*, 1996; Syrjänen, 2002; Guo *et al.*, 2012). Due to the high number of EC cases in Central Asia (100/100,000) in relation to North America and Western Europe (5-10/100,000), the region is known as the Asian Esophageal Cancer Belt (Nasrollahzadeh *et al.*, 2015). Although the reason for EC incidence variation is unknown (Syrjänen, 2002; Nasrollahzadeh *et al.*, 2015), studies indicate the contribution of environmental factors, in addition to infectious agents, in the oncogenic process (Chang *et al.*, 1992; Antonsson *et al.*, 2010).

Smoking and alcohol consumption are pointed out as etiological factors for human EC (Han *et al.*, 1996; Lagergren *et al.*, 1999; Syrjänen, 2002; Haster and Owyang, 2013). However, due to sociocultural reasons, smoking does not justify the high incidence of EC in Central Asia (Nasrollahzadeh *et al.*, 2015), reinforcing the contribution of infectious agent in esophageal carcinogenesis, and evidence shows the etiopathogenic contribution of different infectious agents in EC, such as cytomegalovirus (CMV), Epstein-Barr virus (EBV), herpes simplex and HPV (Syrjänen, 2002).

Although the association between HPV and pre-malignant cervical lesions is known since the 1970s (Syrjänen, 2002), the participation of this virus in EC had its history marked by controversial reports in the literature (Lavergne and de Villiers, 1999). The association between HPV and EC was first proposed by Syrjänen (1982). On the one hand, HPV protein expression was observed in samples by IHC (Winkler *et al.*, 1985; Hille *et al.*, 1986; Kulski *et al.*, 1986) and viral DNA sequences were identified by CISH in EC (Chang *et al.*, 1993; Togawa *et al.*, 1994; Cooper *et al.*, 1995),. On the other hand, serological studies contested these results (Dillner *et al.*, 1995; Han *et al.*, 1996; Lagergren *et al.*, 1999). However, despite these controversial results, the association between HPV and EC is currently accepted, being discussed in the 18^th^ edition of Harrison's Principles of Internal Medicine (Kahrilas and Hirano, 2013). Thus, the absence of HPV detection by serological methods reflects a problem still observed nowadays: the use of antibodies against late (L1) proteins (Dillner *et al.*, 1995; Lagergren *et al.*, 1999). However, considering that EC does not present an HPV productive infection, the late protein expression is not expected. This occurs because HPV presents a “hit and run” mechanism (Han *et al.*, 1996; Bjørge *et al.*, 1997).

Not only serological studies are controversial. Studies based on PCR also show a low correlation between HPV and EC, mainly in clinical samples from Australia (Antonsson *et al.*, 2010; Antonsson *et al.*, 2016). These results can be attributed to the origin of clinical samples, since Australia is not considered a high-risk area for HPV-associated EC. In addition, problems involving primer sensitivity are extensively discussed in the literature of both BPV and HPV (Lavergne and de Villiers, 1999; Silva *et al.*, 2013b; de Villiers, 2013; Araldi *et al.*, 2014b). However, evidence of PVs etiopathogenic action in EC has accumulated along the last 20 years. Among these are: (1) the presence of koilocytes in EC biopsies (Bjørge *et al.*, 1997; Syrjänen, 2002; Vieira *et al.*, 2013), (2) loss or mutation in p53 as a consequence of the PV E6 oncoprotein (Chang *et al.*, 1994, 1995; Herbster *et al.*, 2012), (3) increase in telomerase activity (Syrjänen, 2002), and detection of DNA sequences of HPV-6, 11, 18, 31 and 33 in EC (Vieira *et al.*, 2013).

## Prophylactic and therapeutic vaccines against BPV and HPV

Currently, there are few forms of treatment against BP available (Muro *et al.*, 2008). Among the possibilities is the papilloma surgical excision (Muro *et al.*, 2008). Although frequently employed, this method is inefficient in cattle with high incidence of BPV, because it is impracticable to perform the excision of papillomas of all cattle. Another strategy frequently employed is self-hemotherapy (Leto *et al.*, 2011). This method consists in the removal and intramuscular reinjection of a volume of 10 mL of venous blood, inducing a nonspecific immune stimulus that can promote the “shedding of the warts” (Leto *et al.*, 2011). However, this technique does not avoid BP re-occurrence, thus being a palliative method. Another possibility to reduce the incidence of BP is to control ectoparasite populations, since it was demonstrated that the biological control of ticks reduces the incidence of BPV (William *et al.*, 1992).

Few medical interventions proposed in the last century can match the effects that immunization exerts on longevity (Schuchat and Jackson, 2013). For this reason, vaccination is considered the best form of prevention, control and eradication of viral etiology diseases (Ribeiro-Müller and Müller, 2014). Moreover, immunization reduces both transmission and dissemination of the infectious agent (Schuchat and Jackson, 2013).

Two prophylactic vaccines against HPV are available in the market since 2006: (1) Cervarix, produced by Glaxo-Smith Klein (GSK) and (2) Gardasil, produced by Merck (Ribeiro-Müller and Müller, 2014). These vaccines are based on virus-like particles (VLPs) of the L1 structural protein (Marigliani *et al.*, 2012). Cervarix is a bivalent vaccine able to confer protection against HPV-16 and 18, employing L1 VLPs produced in Baculovirus in *Trichoplusia ni* insect cells, using aluminum hydrophosphate as adjuvant (Ribeiro-Müller and Müller, 2014). Gardasil is able to confer protection against two high-risk HPV types (HPV-16 and 18) and two low-risk ones (HPV-6 and 11), associated to genital warts. This vaccine is composed by of L1 VLPs produced in *Saccharomyces cerevisae*, employing lipid A 3-O-diacilete-44-monophosphoryl (ASO4) as adjuvant‥ Both vaccines are considered safe and well tolerated (Ribeiro-Müller and Müller, 2014). For these reasons, more than 30 countries, including Brazil, adopted immunization programs against HPV based on these vaccines.

Australia, the first country to adopt the vaccination against HPV, observed a reduction of 70% in HPV-6, 11, 16 and 18 infection incidence. Similar results were also verified in Denmark, Finland and Sweden (Ribeiro-Müller and Müller, 2014). However, available vaccines are not able to protect against all HPV types, since there are more than 200 described. Moreover, these vaccines have a high cost of production. Thus, it is necessary to invest in novel multivalent vaccines, with lower production cost. Vaccines based on recombinant protein expressed in *Escherichia coli* have demonstrated to be a useful alternative, because they have a lower cost, and not requiring the L1 VLPs, they are more stable (Ribeiro-Müller and Müller, 2014).

Although there are two vaccines against HPV, there is no vaccine yet against BPV available to date. The idea of developing a vaccine to combat BPV began with the infection of Shope papillomas extract in the 1940 decade (Shope, 1937). Since then, different vaccine models were proposed (Jarret *et al.*, 1991; Gaukroger *et al.*, 1996; Góes *et al.*, 2008; Love *et al.*, 2012; Mazzuchelli-de-Souza *et al.*, 2013). However, none of them became a commercial product. This denotes the notorious difficulty to obtain a safe and efficient vaccine, and reflects the reduced number of research groups dedicated to develop a vaccine against BPV.

Studies have demonstrated that BPV early proteins (E6 and E7) show a therapeutic action, while later proteins (L1 and L2) have a prophylactic action (Campo, 1997b). Vaccines based on E6 and E7 have also been discussed against HPV (Borysiewicz *et al.*, 1996; He *et al.*, 2000; Yao *et al.*, 2013), reinforcing the usefulness of the BPV model not only for comprehending the pathogenic mechanisms of HPV, but also for vaccine biotechnology. However, in a current study based on BPV-1 E6 recombinant oncoprotein, we demonstrated that this oncoprotein is able to induce clastogenesis and neosis *per se* (Araldi *et al.*, 2015a). This data emphasizes the necessity of an *in silico* analysis of E6 and E7 oncoproteins, aiming to obtain novel variants that are more antigenic and less mutagenic. In order to guarantee the immunization and safety of products, our group is now focusing on the development of a prophylactic vaccine based on L1 recombinant protein (Módolo *et al.*, data not published).

## Conclusions

*Papillomaviridae* comprises the most extensive known family of viruses, able to infect all vertebrates including humans, in which it is responsible for 27-30% of all infectious agent-associated incident cancer cases. Moreover, PVs represent an important problem in the veterinary field, inducing papillomas in dogs, felines and cattle. Also, BPV infects equines, resulting in sarcoids. Although novel discoveries contributed to the understanding of the PVs oncogenic role, some carcinogenic mechanisms remain unknown, especially those following cancer initiation (Araldi *et al.*, 2016). In addition, current studies have collected evidence of BPV productive infection in sites earlier considered as not permissive, such as PBMCs (Roperto *et al.*, 2011; Melo *et al.*, 2015), placenta (Roperto *et al,.* 2012) and primary cell cultures (Campos *et al.*, 2008; Campos *et al.*, 2013). Similar results have also been described for HPV (Chiou *et al.*, 2003; Foresta *et al.*, 2013; Pessoa, 2014). However, despite of these advances, the natural history of PVs remains dependent on cell differentiation, emphasizing the need to review the replication cycle of these viruses (White and Howley, 2013; Munday, 2014). In addition, the available vaccines against HPV do not confer protection against all virus types, providing only a limited protection. The veterinary field lives a most dramatic scene, since there is no vaccine available against BPV. Over the last 30 years, our group has dedicated efforts to elucidate the pathogenic mechanisms of PVs, focusing on BPV, once the virus is considered a prototype for HPV studies. Although our contributions brought important advances, more studies are necessary to propose efficacious and safe prophylactic and therapeutic measures.

## Dedication

This paper is dedicated to Dr. Maria Luiza Beçak on occasion of her 80^th^ birthday. She and her husband were the founders of the Laboratory of Genetics of the Instituto Butantan, in 1961. They were Brazil's pioneers in introducing human and vertebrate cytogenetics and the first to use cytogenetics as an important tool in human genetic counseling. Dr. Beçak's contribution in classic and recent papers in the genetic literature is very important and remarkable.
